# Tumor origin and growth pattern at diagnosis and surgical hypothalamic damage predict obesity in pediatric craniopharyngioma

**DOI:** 10.1007/s11060-013-1128-0

**Published:** 2013-04-12

**Authors:** Seung Wan Park, Hae Woon Jung, Young Ah Lee, Choong Ho Shin, Sei Won Yang, Jung-Eun Cheon, In-One Kim, Ji Hoon Phi, Seung-Ki Kim, Kyu-Chang Wang

**Affiliations:** 1 Division of Endocrinology and Metabolism, Department of Pediatrics, Seoul National University Children’s Hospital, Seoul National University College of Medicine, 101 Daehak-ro, Jongno-gu, Seoul, 110-769 South Korea; 2 Department of Radiology, Seoul National University Children’s Hospital, Seoul National University College of Medicine, Seoul, South Korea; 3 Department of Neurosurgery, Seoul National University Children’s Hospital, Seoul National University College of Medicine, Seoul, South Korea

**Keywords:** Craniopharyngioma, Hypothalamus, Obesity, Pediatric

## Abstract

Severe obesity is a major problem in pediatric craniopharyngioma. We investigated whether tumor origin, growth pattern, and surgical damage predict obesity in pediatric craniopharyngioma. Subjects were 58 patients (30 males) with no tumor recurrence during the first postoperative 18 months. Preoperative hypothalamic involvement was classified into no (pre_G0, *n* = 19), little (pre_G1, *n* = 21), and severe (pre_G2, *n* = 18) involvement groups based on sub- or supradiaphragmatic tumor origin and growth patterns. Postoperative hypothalamic involvement was classified into no (post_G0, *n* = 4), minimal (post_G1, *n* = 19), and significant (post_G2, *n* = 35) involvement groups according to follow-up imaging. The prevalence of obesity increased from 13.2 % at diagnosis (mean age = 8.1 years) to 37.9 % at last follow-up (mean duration = 9.1 years). Only the body mass index (BMI) Z-score increment of the first postoperative year (first-year ΔBMI_Z) was significant (*P* = 0.007). Both the preoperative BMI_Z (*P* = 0.001) and the first-year ΔBMI_Z (*P* = 0.017) showed an increasing trend from the pre_G0 to pre_G1 to pre_G2 group. For the 40 patients with pre_G0 or pre_G1, the first-year ΔBMI_Z was higher in the post_G2 group than the post_G1 group (0.02 ± 0.91 vs. 0.89 ± 0.72, *P* = 0.003). Tumor origin and growth pattern affect preoperative BMI_Z and postoperative weight gain. Despite little or no hypothalamic involvement at diagnosis, surgical damage contributes to postoperative weight gain in patients with craniopharyngioma.

## Introduction

Childhood craniopharyngiomas are sellar and/or parasellar tumors originating from the remnants of Rathke’s pouch. Although histologically benign, they are clinically malignant because of their anatomical proximity to the optic nerves, pituitary gland, and hypothalamus, and may cause visual impairment, endocrine deficiencies, and obesity after tumor resection surgery [1]. The most notable adverse effect is severe obesity, which has a major negative impact on cardiovascular health and the quality of life of long-term survivors [2–4].

Obesity rates in children with craniopharyngiomas vary from 6 to 30 % at diagnosis and increase to 40–60 % after surgery [1, 3, 5, 6]. Higher body mass index Z-scores (BMI_Z) at the time of diagnosis and early and rapid postoperative weight gain are the greatest risk factors of future obesity [6–9]. A major possible cause of obesity in these children is the disruption of hypothalamic mechanisms that control satiety, hunger, and energy balance [8]. The relationship between pre- and postoperative hypothalamic damage and obesity has been previously noted in the literature, with several studies of Caucasian patients showing an association between hypothalamic damage on magnetic resonance imaging (MRI) and postoperative weight gain and obesity [2, 5, 6, 10, 11]. However, there are few data on the prevalence of obesity, longitudinal changes in BMI_Z, and the relationship between hypothalamic damage and obesity in Asian craniopharyngioma patients.

We hypothesized that the degree of preoperative hypothalamic involvement based on tumor origin and growth pattern in relation to the diaphragm sellae would predict the degree of obesity at diagnosis. We also investigated changes in the anthropometric characteristics during 4 years of follow-up and considered whether preoperative hypothalamic involvement and surgical damage were predictive of postoperative changes in BMI_Z during the critical period of rapid weight gain.

## Methods

### Patients

This study was approved by the Seoul National University Hospital Institutional Review Board. Medical records of craniopharyngioma cases operated on between July 1987 and May 2010 at Seoul National University Children’s Hospital were retrospectively reviewed. During the years reviewed, all operations were aimed at radical excision whenever possible. Between 1987 and 2000, the extended pterional approach was the surgical approach of choice. Between 2000 and 2010, there was a shift toward the interhemispheric subfrontal approach for tumors of supradiaphragmatic origin and the microscopic transsphenoidal approach for tumors of subdiaphragmatic and prechiasmatic origin. Since 2010, we have adopted the endoscopic endonasal approach for pediatric craniopharyngioma, but these patients were not included because of their relatively short follow-up period.

**Fig. 1:**
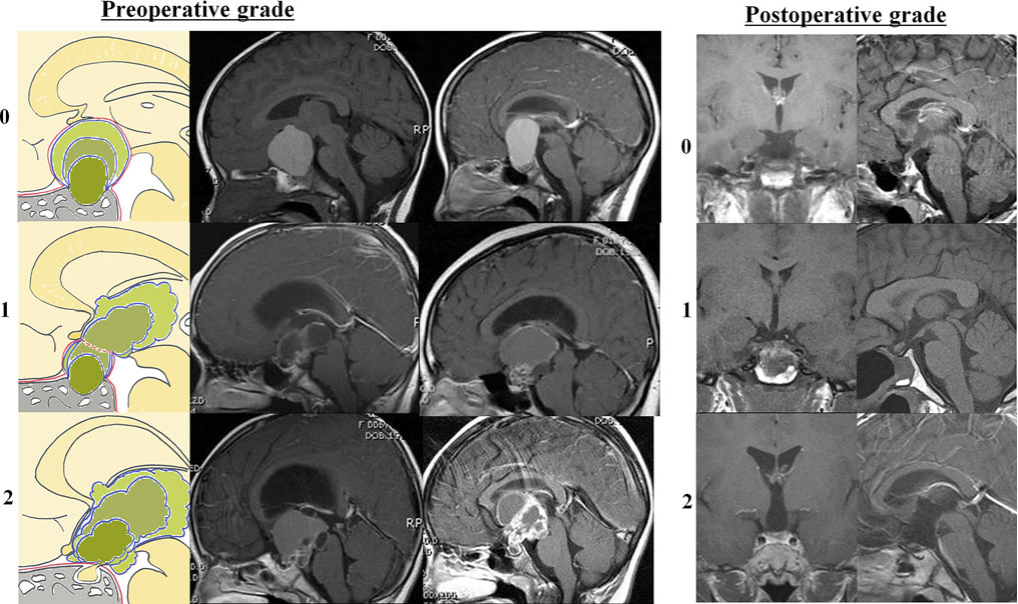
Preoperative (*left column*) and postoperative (*right column*) classification of pediatric craniopharyngiomas. *Upper left* Grade 0 (pre_G0, no hypothalamic involvement), a tumor of subdiaphragmatic origin with competent diaphragm sellae, which grows in the prechiasmatic direction. *Center left* Grade 1 (pre_G1, little hypothalamic involvement), a tumor of subdiaphragmatic origin with incompetent diaphragm sellae, which grows the diaphragmatic aperture in the retrochiasmatic direction. *Lower left* Grade 2 (pre_G2, severe hypothalamic involvement), a tumor of supradiaphragmatic origin, which grows retrochiasmatically in the direction of the third ventricular floor or into adjacent cerebrospinal fluid spaces (reprinted from *Childs Nerv Syst (2005) 21: 628*–*634* with permission from Elsevier). *Upper right* Grade 0 (post_G0), no hypothalamic damage. *Center right* Grade 1 (post_G1), minimal hypothalamic damage. *Lower right* Grade 2 (post_G2), significant hypothalamic damage

According to the tumor origin and growth patterns, which were based on preoperative MRI and/or operative findings, the *preoperative hypothalamic involvement* was graded as follows: grade 0 (pre_G0), a tumor of subdiaphragmatic origin with competent diaphragm sellae growing in the prechiasmatic direction; grade 1 (pre_G1), a tumor of subdiaphragmatic origin with incompetent diaphragm sellae growing through the diaphragmatic aperture in the retrochiasmatic direction; and grade 2 (pre_G2), a tumor of supradiaphragmatic origin growing retrochiasmatically in the direction of the third ventricular floor or into adjacent cerebrospinal fluid spaces (Fig. 1) [12]. All patients underwent postoperative brain MRI follow-up at 3 and 6 months, and annually thereafter. Tumor recurrence and progression were defined as the reappearance of, or increase in, contrast enhancement on the postoperative MRI, respectively.

Five patients (1 pre_G0, 1 pre_G1, and 3 pre_G2) who developed tumor recurrence within 18 months were excluded to allow analysis of the effect of preoperative hypothalamic involvement on the first-year postoperative weight gain. Thus, the study included a total of 58 patients (30 males). Of these, 3 were diagnosed between 1987 and 1990, 19 were diagnosed between 1991 and 2000, and 36 were diagnosed between 2001 and 2010. All operations were aimed at total removal whenever possible with the following results: gross total removal (*n* = 48), near total removal (*n* = 6), subtotal removal (*n* = 2), and subtotal removal and radiotherapy (*n* = 2). The surgical approach, either transcranial (*n* = 52) or microscopic transsphenoidal (*n* = 6), was decided on in accordance with tumor origin and growth pattern. The pituitary stalk was preserved in 9 patients. Fifteen of the 58 patients (11 of 48 patients with gross total removal, 3 of 6 patients with near total removal, and 1 of 5 patients with subtotal removal) developed tumor recurrence after 2.5 ± 1.2 years (range = 1.6–5.3 years). These patients were further treated by reoperation and radiotherapy (*n* = 7), reoperation and gamma knife surgery (GKS) (*n* = 3), reoperation, radiotherapy and GKS (*n* = 1), radiotherapy and GKS (*n* = 1), and radiotherapy only (*n* = 3). The total number of operations was as follows: once (*n* = 47), twice (*n* = 7), three times (*n* = 2), and four times (*n* = 2). Based on brain MRI 3 and 6 months after surgery, the *postoperative hypothalamic involvement* was independently graded by a neuroradiologist as follows: grade 0 (post_G0), no hypothalamic involvement; grade 1 (post_G1), negligible hypothalamic involvement or residual tumor displacing the hypothalamus; and grade 2 (post_G2), significant hypothalamic involvement (floor of the third ventricle not identifiable) (Fig. 1) [11]. Only 1 patient from the post_G1 group was reclassified into the post_G2 group, at the last follow-up, because of tumor recurrence.

All patients were preoperatively evaluated for whether thyroid hormone, hydrocortisone, and/or antidiuretic hormone (ADH) needed to be replaced. After surgery, hydrocortisone was tapered to physiologic doses (10–15 mg/m^2^/day) and thyroid hormone was adjusted according to postoperative thyroid function tests. Patients who were postoperatively diagnosed with permanent diabetes insipidus were placed on ADH replacement. All patients underwent pituitary function testing including GH within 1 year of the final tumor treatment. Forty-eight patients had GH, ACTH, TSH, and ADH deficiencies and 10 patients were deficient in three of these four hormones (Table 1). High dose GH for statural growth (0.5–0.7 mg/m^2^/week) or low dose GH for metabolic effect (0.1 mg/m^2^/week) was started approximately 1 year after the final tumor treatment and was continued for more than 1 year in 45 patients. The remaining 13 patients did not receive GH therapy because the Korean medical insurance policy did not cover this therapy in females over 150 cm or males over 160 cm, or the patients were discontinued from GH therapy within 6 months because of tumor recurrence and/or side effects of GH therapy. Except for some of the patients who did not complete GH therapy, all patients were under good hormonal control during follow-up.

**Table 1:** Comparison of clinical characteristics of patients at three levels of preoperative hypothalamic involvement based on tumor origin and growth pattern in relation to diaphragmatic sellae

	Preoperative hypothalamic involvement			*P* value
	Grade 0	Grade 1	Grade 2	
N (males:females)	19 (7:12)	21 (12:9)	18 (11:7)	0.141
Age at diagnosis (years)	7.8 ± 3.4	8.8 ± 3.1	7.7 ± 3.8	0.575
Obesity at diagnosis [N (%)]	0 (0)	2 (9.5)	6 (33.3)	0.031
N (postoperative grade 0:1:2)	19 (4:12:3)	21 (0:5:16)	18 (0:3:15)	<0.001
Obesity 1 year after surgery [N (%)]	1 (5.3)	4 (19)	12 (66.7)	<0.001
Age at last evaluation (years)	17.5 ± 6.9	19.9 ± 7.3	16.8 ± 4.6	0.280
Obesity at last evaluation [N (%)]	2 (10.5)	10 (47.6)	10 (55.5)	0.001
Duration of follow-up (years)	8.8 ± 4.6	9.8 ± 5.3	8.5 ± 4.1	0.653
Height Z-score at last evaluation	−0.03 ± 1.91	−0.06 ± 1.33	0.37 ± 1.10	0.613
Weight Z-score at last evaluation	−0.23 ± 1.62^*,†^	0.97 ± 1.35^†^	1.51 ± 1.04^*^	0.001
BMI Z-score at last evaluation	−0.14 ± 0.83^*,†^	1.10 ± 1.18^†^	1.49 ± 1.08^*^	<0.001
Change in BMI Z-score (from diagnosis to last evaluation)	−0.17 ± 1.27	0.82 ± 1.39	0.50 ± 1.17	0.053
Relapse (N)	6	6	3	0.307
Number of operations (1:2:≥3)	14:4:1	17:3:1	16:0:2	0.689
Radiotherapy (N)	4	6	1	0.243
Gamma knife surgery (N)	2	2	1	0.596
Life stage at first GH treatment [N (childhood: adulthood: not started yet)]	13:1:5	17:1:3	11:2:5	0.791
Number of hormone deficiencies (2:3:4)^‡^	1:8:10	2:6:13	0:6:12	0.328
Central diabetes insipidus [N (%)]	17 (89.4)	19 (90.5)	17 (94.4)	0.841

Data are expressed as mean ± SD

Analysis of variance models were used to compare continuous variables between the three groups according to preoperative hypothalamic involvement. The difference in the means of variables within two subsets was analyzed using the Bonferroni method with *P* set at 0.025 [^*^ *P* < 0.01, preoperative grade 0 vs. preoperative grade 2] and [^†^ *P* < 0.025, preoperative grade 0 vs. preoperative grade 1]. The comparison of categorical variables between the two (three) groups was analyzed by the Chi squared test (Chi squared test for trend)

*BMI* body mass index, *GH* growth hormone

^‡^Number of the following pituitary hormone deficiencies: GH, ACTH, TSH, and ADH

Height (cm) was measured twice with a Harpenden stadiometer (Haltain Ltd, Crymych, Wales, UK), and weight (kg) was measured to the first decimal place with a digital scale. Height Z-score (height_Z), weight Z-score (weight_Z), body mass index Z-score (BMI_Z) and BMI percentile were assigned using the 2007 Korean National Growth Charts [13], and patients were classified into lean (<85th BMI percentile; BMI_Z < 1.04), overweight (85–95th BMI percentile; 1.04 to <1.65 of BMI_Z), and obese (≥95th BMI percentile; BMI_Z ≥ 1.65) groups.

### Statistical analysis

Statistical analyses were performed using SPSS for Windows (version 18.0, SPSS Institute, Chicago, IL). All continuous variables were described as the mean ± SD. Student’s *t* tests were performed to compare the means of two independent variables. Paired *t* tests were used to compare the means of two related variables. To compare height_Z, weight_Z, and BMI_Z between the three groups according to hypothalamic involvement, analysis of variance was used. The difference in the means of variables within two subsets was analyzed using the Bonferroni method with *P* set at 0.025. A Chi squared test was used for trend analysis to compare the proportion of categorical variables between the three groups. The significance of an increasing trend of preoperative BMI_Z and the first-year increase in BMI_Z with increasing hypothalamic involvement from the pre_G0 to pre_G1 to pre_G2 group was assessed using linear regression analysis. Univariate linear regression analysis was performed to identify predictors for BMI_Z 4 years after surgery. Multivariate regression analysis was subsequently performed including all independent variables significant in the univariate analysis and previously known covariates. For all analyses, *P* < 0.05 (two-sided) was considered statistically significant.

## Results

### Preoperative and postoperative hypothalamic involvement classification and anthropometric changes during 4 year follow-up

The 58 patients (30 males, 8.1 ± 3.5 years of age) were classified as being lean (*n* = 41, 71.7 %), overweight (*n* = 9, 15.5 %), or obese (*n* = 8, 13.8 %) at diagnosis. They were reclassified at their last follow-up (age = 18.1 ± 6.5 years, duration = 9.1 ± 4.6 years) as being lean (*n* = 32, 55.2 %), overweight (*n* = 4, 6.9 %), or obese (*n* = 22, 37.9 %). The last BMI_Z of those 43 patients who had achieved adulthood by their last follow-up was 1.01 ± 1.25, with 6 (14 %) patients being overweight and 16 (37.2 %) patients being obese.

**Fig. 2:**
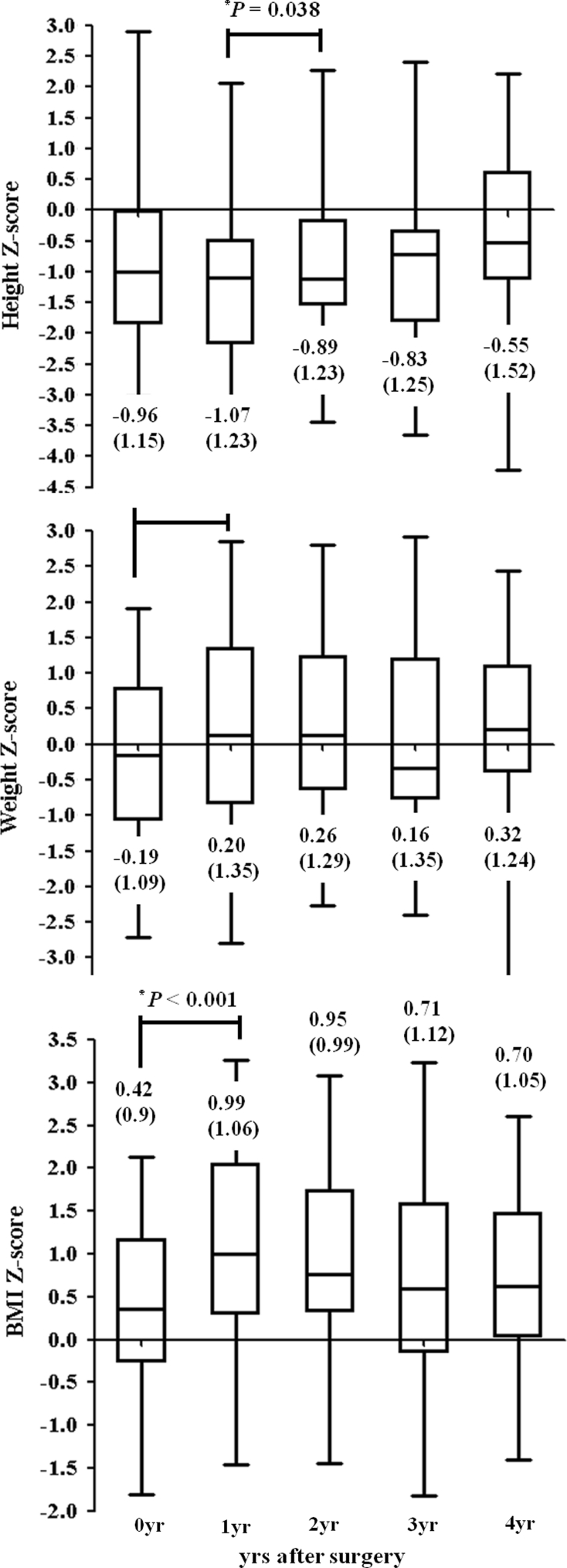
Changes in height, weight, and BMI Z-scores during the 4 years of follow-up. The Z-scores (±SD) are indicated at the *bottom* of each column

Thirty-eight patients reached the 4 year follow-up. During these 4 years, only the increases in weight_Z (*P* = 0.007) and BMI_Z (*P* = 0.007) during the first postoperative year were significant (Fig. 2). Univariate regression analysis showed that BMI_Z 4 years after surgery was significantly predicted by both preoperative (*P* = 0.04) and postoperative (*P* < 0.001) hypothalamic involvement and the BMI_Z at diagnosis (*P* = 0.05). It was unaffected by sex, age at diagnosis, gross total removal, pituitary stalk preservation, tumor recurrence, reoperations, radiotherapy, number of hormone deficiencies (among ACTH, TSH, GH), central diabetes insipidus, and GH treatment. In the multivariate regression analysis, the grade of postoperative hypothalamic involvement (*P* = 0.016) was the only significant predictor related to BMI_Z 4 years after surgery, after adjusting for preoperative hypothalamic involvement, BMI_Z at diagnosis, and other known variables (adjusted *R* ^2^ = 0.25).

### Tumor origin and growth pattern determine the degree of obesity at diagnosis and the first-year increase in BMI_Z after surgery

**Fig. 3:**
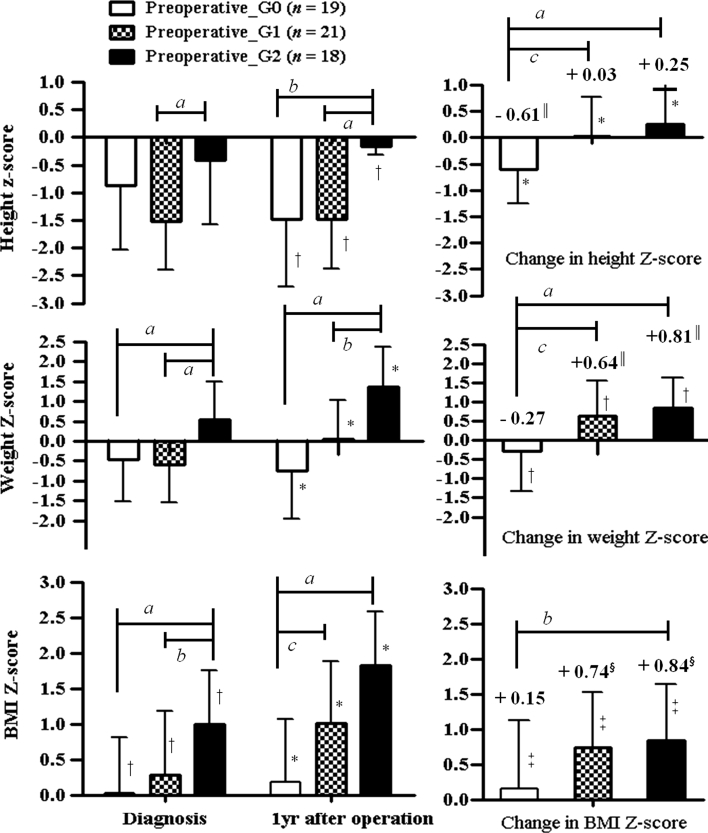
Height, weight, and BMI Z-scores at diagnosis and 1 year after operation (*left column*) and first-year changes in height, weight, and BMI Z-scores (*right column*). The difference in the means of variables within two subsets was analyzed using the Bonferroni correction for multiple comparisons with *P* set at 0.025 (^a^ *P* < 0.01, vs. preoperative_G2 group, ^b^ *P* < 0.025, vs. preoperative_G2 group, ^c^ *P* < 0.025 [preoperative_G0 vs. preoperative_G1 group]). The significance of the increasing trend of height, weight, and BMI Z-scores at diagnosis and 1 year after operation and the first-year changes in height, weight, and BMI Z-scores with increasing hypothalamic involvement was analyzed using linear regression analysis (^*^ *P* < 0.001, ^†^ *P* < 0.01, and ^‡^ *P* < 0.05). The significance of changes in height, weight, and BMI Z-scores during the first year in each group was analyzed using paired *t* tests (^§^ *P* < 0.001, and ^║^ *P* < 0.01)

Patients were classified into pre_G0 (*n* = 19), pre_G1 (*n* = 21), and pre_G2 groups (*n* = 18) according to preoperative hypothalamic involvement (Fig. 1). Greater preoperative hypothalamic involvement was associated with greater postoperative hypothalamic involvement (*P* < 0.001) (Table 1). The BMI_Z at the time of diagnosis increased with each progressive grade of hypothalamic involvement (0.03 ± 0.79 for pre_G0 vs. 0.28 ± 0.91 for pre_G1 vs. 0.99 ± 0.76 for pre_G2, *P* < 0.001) (Fig. 3, left column). The proportion of obese patients also increased significantly from the pre_G0 (0 %) to pre_G1 (9.5 %) to pre_G2 group (33.3 %) (*P* = 0.03, Table 1). There was no difference in age or sex distribution in the three groups. Height_Z at diagnosis was significantly higher in pre_G2 patients than in pre_G1 group (−0.41 ± 1.17 vs. −1.51 ± 0.89, *P* = 0.002), but not when compared with pre_G0 group patients. The postoperative first-year increase in BMI_Z showed progressively higher levels of hypothalamic involvement from the pre_G0 (0.15 ± 0.97) to the pre_G1 (0.74 ± 0.79) to the pre_G2 group (0.84 ± 0.80) (*P* = 0.017). The BMI_Z of the pre_G0 group did not change significantly, but those of the pre_G1 and pre_G2 groups increased significantly during the first postoperative year (*P* < 0.001 for both) (Fig. 3, right column).

### Despite little or no hypothalamic involvement at diagnosis, surgical hypothalamic damage predicts the first-year increase in BMI_Z

Postoperative hypothalamic involvement was classified into post_G0 (no involvement, *n* = 4), post_G1 (*n* = 20), and post_G2 groups (*n* = 34) (Fig. 1). The first-year increase in BMI_Z of the post_G2 group was higher than that of the post_G1 group (0.16 ± 0.99 vs. 0.84 ± 0.77, *P* = 0.007).

**Fig. 4:**
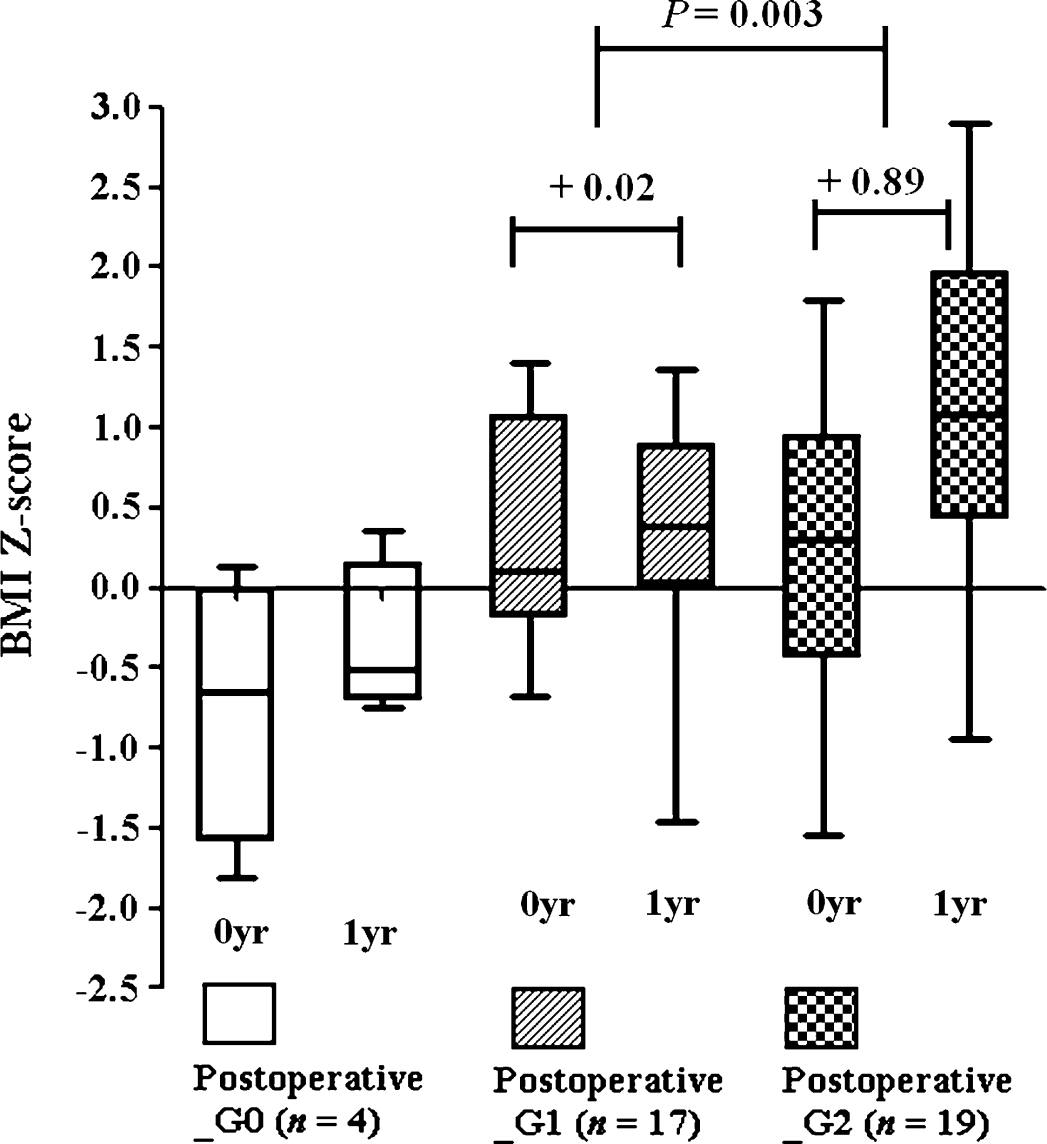
The first-year increase in BMI Z-scores of the postoperative_G2 group (+0.89) was higher than that of the postoperative_G1 group (+0.02) in an analysis of 40 patients who had little or no hypothalamic involvement at diagnosis (*P* = 0.003)

Forty patients initially classified into the pre_G0 or pre_G1 groups at diagnosis were postoperatively classified as post_G0 (*n* = 4), post_G1 (*n* = 17), or post_G2 (*n* = 19) (Table 1). When these 40 patients were analyzed, the first-year increase in BMI_Z of the post_G2 group was higher than that of the post_G1 group (0.02 ± 0.91 vs. 0.89 ± 0.72, *P* = 0.003), showing that additional surgical damage is significantly related to the first-year increase in BMI_Z (Fig. 4).

## Discussion

The prevalence of obesity at the time of diagnosis was 13.2 % and had increased to 37.9 % by the last follow-up. During the 4 years of follow-up, the first postoperative year was the critical period of rapid weight gain. Both the preoperative BMI_Z and the first-year increase in BMI_Z showed an increasing trend from the group without hypothalamic involvement to those with progressively more severe involvement. In patients classified with little or no hypothalamic involvement at the time of diagnosis, additional surgical damage resulted in a greater increase in BMI_Z during the first postoperative year.

At the time of diagnosis, none of the patients classified as having no initial hypothalamic involvement were obese, while one-third of the patients with severe hypothalamic involvement were obese. Patients with severe hypothalamic involvement were not only obese, but also unexpectedly taller than those classified into the groups of no or low hypothalamic involvement, an indication that the high BMI in those with severe hypothalamic involvement was not caused by short stature. Despite GH deficiency, the mechanism that maintains the normal height gain that accompanies weight gain in patients with severe hypothalamic involvement is not clear. Normal growth in spite of GH deficiency may be explained by a complex series of metabolic events including activation of insulin-like growth factor I by hypothalamic hyperphagia and/or obesity-induced hyperinsulinism [14].

Previous classification systems in other studies were either able to show a significant relationship between preoperative hypothalamic involvement and the degree of obesity at diagnosis [5, 7, 15] or not [2]. Our preoperative assessment of hypothalamic involvement was based on tumor origin and growth pattern in relation to the diaphragmatic sellae. When the tumor originates below the level of the diaphragm sellae, the tumor will elevate and stretch the diaphragm sellae upward. If the tight sealing of the diaphragm sellae and the dura mater of the skull base limit posterior growth of the tumor in the direction of the third ventricle, the tumor may remain confined subdiaphragmatically without hypothalamic involvement. However, if the tumor leaks through the opening, it will have direct contact with the suprasellar structures. The incomplete diaphragm will constrict the tumor during its growth, resulting in a snowman-like mass composed of upper supradiaphragmatic and lower subdiaphragmatic parts with little hypothalamic involvement. On the other hand, supradiaphragmatic tumors, and especially the cystic contents of the tumor, can grow unrestrained to the third ventricle with extensive adhesion to adjacent structures and severe hypothalamic involvement (Fig. 2) [12]. Our preoperative hypothalamic involvement grades were significantly related to the degree of obesity at diagnosis. Although we could not investigate changes in BMI_Z before diagnosis, preoperative hypothalamic involvement by tumor origin and growth pattern may change appetite and energy expenditure and cause pituitary hormone deficiencies, leading to progressive weight gain.

Morbid obesity in relation to hypothalamic lesions is poorly responsive to diet, physical activity, and most pharmacological therapies [9, 16]. Thus, early prophylactic and therapeutic interventions are recommended during the critical period of rapid weight gain [17]. In our study, the first postoperative year was the critical period for rapid increase in BMI_Z, which was predicted by pre- and postoperative hypothalamic involvement. Most patients with craniopharyngioma experience a major increase in weight and BMI during the early postoperative period [9, 15]. Children who develop hypothalamic obesity experience a significant, rapid increase in BMI over the first 6 months [10, 18], followed by stabilization without regression of the BMI_Z [18]. Perioperative dexamethasone administration [19], GH deficiency [20], and surgical hypothalamic damage may contribute to early postoperative weight gain. In the 40 patients classified as having little or no hypothalamic involvement at diagnosis, additional hypothalamic damage after surgery resulted in a greater increase in first-year BMI_Z. Postoperative hypothalamic involvement predicted BMI_Z 4 years postoperatively, independent of age, BMI_Z, and preoperative hypothalamic involvement at diagnosis. Clearly, surgical strategies to preserve hypothalamic integrity by expert surgeons are mandatory for the prevention of severe obesity caused by hypothalamic lesions. Müller et al. [2] recently recommended that treatment should be confined to experienced multidisciplinary teams because patients treated with radical resection in small-sized centers present with a higher rate of hypothalamic involvement and a greater negative impact on quality of life than those treated in medium- and large-sized centers. In addition, early changes to lifestyle should be emphasized during the early postoperative period, especially in patients with greater hypothalamic damage.

This retrospective study was limited by the fact that we could not investigate diet, physical activity, daytime somnolence, and autonomic nervous dysfunction related to hypothalamic dysfunction, and other markers of adiposity, such as total or visceral body fat. In addition, our study, which included gross total resection attempts whenever possible, could not answer the controversial issue regarding unfavorably localized hypothalamic tumors (complete resection versus a planned limited resection and adjuvant therapy). With radical resection, there is a risk of surgically induced hypothalamic damage related to severe obesity and poor quality of life. With incomplete resection followed by irradiation, concerns about residual tumor relapse and/or risk of irradiation-associated secondary malignancy may result in delays or hesitancy in implementing GH therapy [21]. Although no significant relationship between GH therapy and BMI_Z 4 years after surgery was found in our study, exposure to long-term GH deficiency may increase visceral adiposity and cardiovascular morbidity [8]. For direct evidence of how hormonal changes affect postoperative adiposity, analyses of longitudinal changes in adiposity before and after the start of GH therapy are needed. In our study, although yearly changes in BMI_Z were retrospectively reviewed with respect to the start of GH therapy, there was too much missing data for a complete statistical analysis. Future studies of changes in adiposity with respect to GH therapy are needed. Prospective longitudinal studies examining ideal treatment modalities for unfavorably localized craniopharyngioma and effective interventional trials for preventing progressive obesity are required. The benefit of recently adopted endoscopic endonasal surgery, which aims to maximize tumor resection with minimal surgical morbidity and hypothalamic injury, also needs to be addressed in further studies.

In conclusion, tumor origin and growth pattern predicted the degree of obesity at diagnosis and the first year postoperative weight gain. Despite little or no hypothalamic involvement at diagnosis, surgical damage resulted in a greater increase in BMI_Z during the first postoperative year. Our study underlines the role of experienced surgeons in minimizing surgical hypothalamic damage. In cases when the tumor does involve the hypothalamus, an attempt at complete resection to cure the patient should be balanced with quality of life. All patients, especially those with hypothalamic injury, should be informed of the risk of progressive obesity and the importance of early intensive lifestyle interventions during the first postoperative year and subsequent follow-up.
